# A Bayesian network meta-analysis: Comparing the clinical effectiveness of local corticosteroid injections using different treatment strategies for carpal tunnel syndrome

**DOI:** 10.1186/s12891-015-0815-8

**Published:** 2015-11-19

**Authors:** Po-Cheng Chen, Ching-Hui Chuang, Yu-Kang Tu, Chyi-Huey Bai, Chieh-Feng Chen, Mei- Yun Liaw

**Affiliations:** Department of Rehabilitation, Kaohsiung Chang Gung Memorial Hospital, Kaohsiung, Taiwan; Department of Nursing, Kaohsiung Chang Gung Memorial Hospital, No.123, Dapi Road, Niaosong District, Kaohsiung, 83301 Taiwan; Institute of Epidemiology and Preventive Medicine, College of Public Health, National Taiwan University, Taipei, Taiwan; School of Public Health, Taipei Medical University, Taipei, Taiwan; Division of Plastic Surgery, Department of Surgery, Wan Fang Hospital, Taipei Medical University, Taipei, Taiwan; Cochrane Taiwan, Taipei Medical University, Taipei, Taiwan; School of Nursing, Chang Gung University of Science and Technology, Chiayi, Taiwan

**Keywords:** Network meta-analysis, Local corticosteroid injection, Carpal tunnel syndrome

## Abstract

**Background:**

Local corticosteroid injections are commonly used to improve the short-term symptomatic severity and the functional status of the hands affected by carpal tunnel syndrome. We conducted a systematic review and Bayesian network-meta-analysis to compare the clinical effectiveness of local corticosteroid injections using different injection approaches.

**Methods:**

Electronic literature in Cochrane Central Register of Controlled Trials (CENTRAL), MEDLINE, EMBASE, Web of Science, and other sources were searched to identify clinical studies comparing different injection approaches with each other or placebo for carpal tunnel syndrome. Two review authors conducted selection of studies, data extraction, and assessment of risk of bias independently. Random-effects models were used to conduct the pairwise meta-analysis and the Bayesian network meta-analysis.

**Results:**

Overall, 10 studies with 633 patients were included in the systematic review. Among the injection approaches, local corticosteroid injections using the ultrasound-guided in-plane injection (Ulnar-I) approach was the best treatment strategy for clinical response (median OR versus placebo 128.30, 95 % CrI 9.76 to 2299.00), change in symptom severity scale (median MD versus placebo −1.16, 95 % CrI −1.95 to −0.38) , and change in functional status scale (median MD versus placebo −0.74, 95 % CrI −2.00 to 0.52) at short-term follow-up period in the network meta-analysis. Local corticosteroid injections using other injection approaches were better than placebo for clinical response (for the PI approach, median OR versus placebo 8.85, 95 % CrI 3.00 to 33.15; for the DI approach, median OR versus placebo 7.00, 95 % CrI 0.53 to 118.80) , change in symptom severity scale (for the Ulnar-O approach, median MD versus placebo −0.78, 95 % CrI −1.43 to −0.16; for the PI approach, median MD versus placebo −0.58, 95 % CrI −0.95 to −0.22), and change in functional status scale (for the Ulnar-O approach, median MD versus placebo −0.63, 95 % CrI −1.67 to 0.43; for the PI approach, median MD versus placebo −0.46, 95 % CrI −1.11 to 0.21) at short-term follow-up period. The quality of studies is good.

**Conclusions:**

According to our analyses, the ultrasound-guided in-plane injection (Ulnar-I) approach was the most effective treatment among the injection approaches for carpal tunnel syndrome.

## Background

Carpal tunnel syndrome is a common focal peripheral neuropathy caused by compression of the median nerve at the wrist. [1] It results in pain, burning, tingling, or paresthesia in distribution of median nerve distal to the wrist [2, 3] The age distribution is bimodal with first peak in early 50s and second peak at age 75–84 years, and women, especially during pregnancy, are more commonly affected than men [4–6]. In United States, the prevalence of clinically diagnosed carpal tunnel syndrome among workers was 6.7 %, while the prevalence of confirmed carpal tunnel syndrome ranged from 2.7 to 4.9 % in Sweden [7, 8].

There are many causes and risk factors for carpal tunnel syndrome, such as trauma, vascular lesions, inflammation, obesity, occupational exposure, older age, osteoarthritis, pregnancy, hypothyroidism, or autoimmune diseases [2–4, 9, 10]. The pathogenesis may be median nerve compression leading to ischemia of median nerve, which impairs neural conduction and nerve damage [4]. Carpal tunnel syndrome is a clinical diagnosis supported by specific findings on provocative tests, such as Phalen test or Tinel test [11].

The severity of carpal tunnel syndrome can be divided into 5 levels, from very mild symptoms (pins and needles sensation, pain, or sensibility loss in the fingers and/or hand, mostly only during nighttime) to continuously very severe symptoms (pins and needles sensation, pain, significant then atrophy, and/or significant sensibility loss in the fingers and/or hand, most time) [12]. In patients with mild-to-moderate carpal tunnel syndrome, most symptoms will respond to conservative treatment or resolve spontaneously [4]. It is believed that about 34 % of patients with idiopathic carpal tunnel syndrome may have spontaneous improvement [13].

Various interventions have been attempted to treat carpal tunnel syndrome. These interventions can be categorized into two types, surgical and nonsurgical interventions. The surgical interventions include open carpal tunnel release, minimal incision carpal tunnel release, or endoscopic carpal tunnel release [14–16]. The nonsurgical interventions include activity modification, wrist splints, oral medications, local corticosteroid injections, or other managements (such as laser therapy, therapeutic ultrasound, or acupuncture) [17, 18].

Nonsurgical interventions are usually initiated for mild to moderate carpal tunnel syndrome [19]. In most cases, local corticosteroid injection is usually considered before surgery [17]. Because the prevalence of mild to moderate carpal tunnel syndrome is high, the impact of this conservative intervention may be significant for the syndrome.

Local corticosteroid injections at the site of the carpal tunnel may reduce tendon swelling and lead to decompression of the median nerve [17]. The usual injection site is the proximal carpal tunnel near the flexor crease and just ulnar to the palmaris longus tendon. If patients have no palmaris longus tendon, ulnar to midline of wrist or just ulnar to flexor carpi radialis tendon is another choice [20–22]. The distal injection approach, in which the injection site is distal to the middle of the flexor crease, was recently used in one study [23]. Ultrasound-guided injection is gradually being adopted for accurate localization and better outcome [24, 25].

Carpal tunnel syndrome is a major disorder bothering the life quality of patients. Additionally, the syndrome interferes with the complex movements and tactile sensation of the hand [17]. Local corticosteroid injection may be the fastest and the most efficient method for improvement of symptoms. Some of the interventions have been systematically reviewed previously, and the results show that they can reduce short-term symptoms prior to definitive surgeries [26]. However, to our knowledge, no review has been conducted to assess the comparative effectiveness of different injection approaches to improve the severity and function of the hands affected by carpal tunnel syndrome. This systematic review is intended as a useful guide for patients to understand the role of different injection approaches in improving the severity and function of the hands affected by carpal tunnel syndrome.

## Methods

The study included randomized placebo-controlled trials of corticosteroid injections for carpel tunnel syndrome or head to head trials evaluating different corticosteroid injections, irrespective of the dose, potency, or duration of corticosteroid, the size of syringes or needles, and angles of needle entry. Participants with mild to moderate degree of carpal tunnel syndrome were included, and there was no restriction in the mean duration of symptoms. We excluded randomized clinical trials in which participants received treatments other than local corticosteroid injections.

### Information sources and search strategy

We included randomized clinical trials that assessed the effect of local corticosteroid injections using different injection approaches compared with each other or with placebo.

There were four main injection approaches for carpal tunnel syndrome.

1. Proximal corticosteroid injection (PI): The injection site is the proximal carpal tunnel near the flexor crease at the wrist.
2. Distal corticosteroid injection (DI): The injection site is distal to the middle of the flexor crease at the wrist.
3. Ultrasound-guided in-plane injection (Ulnar-I): The needle enters the skin at the side of the transducer. The needle traverses the plane of ultrasound and the whole shaft is visualized as it progresses towards the target.
4. Ultrasound-guided out-plane injection (Ulnar-O): The needle enters the skin away from the transducer, and it is aimed at the plane of sound. With this approach, just the needle tip is visualized and the remainder of the needle is off screen.

More symptomatic relief could be reported due to more local corticosteroid injection times. To simplify the clinical conditions, we restricted the total injection times to 1 ~ 2 times.

The study defined local corticosteroid injections using different injection approaches as different treatment strategies. The purpose of this review was to identify the overall treatment effect of a treatment strategy rather than the contribution of each component intervention towards the overall effect.

### Types of outcome measures

We assessed the comparative effectiveness of available treatment strategies that aimed to improve the short-term symptomatic severity and the functional status of the hands affected by carpal tunnel syndrome. Clinical response was defined as an asymptomatic hand (VAS < 2 cm), patients’ satisfaction, or patients’ favorable response after injection [23, 27, 28]. Boston Carpal Tunnel Questionnaire was used to evaluate the symptom severity scale (SSS, 11 items, total score = 11 to 55) and the functional status scale (FSS, 8 items, total score = 8 to 40). We defined change in symptom severity scale as SSS at short-term follow-up period minus SSS at baseline (SSS _short-term_ – SSS _baseline_) and change in functional status scale as FSS at short-term follow-up period minus FSS at baseline (FSS _short-term_ – FSS _baseline_). We used the outcomes of clinical response (binary outcome), change in symptom severity scale (continuous outcome), and change in functional status scale (continuous outcome). We used outcomes assessed before or near 8 weeks.

### Search methods for identification of studies

#### Electronic searches

We searched the Cochrane Central Register of Controlled Trials (CENTRAL), MEDLINE, EMBASE, and Web of Science to 31 May 2015. We also searched the World Health Organization International Clinical Trials Registry Platform search portal, which searches various trial registers, including ISRCTN and ClinicalTrials.gov (http://apps.who.int/trialsearch/Default.aspx) to identify further trials. Search strategies are available in Appendix 1. We adopted the search filters developed by the Hedges Project (http://hiru.mcmaster.ca/hiru/HIRU_Hedges_home.aspx) to achieve best balance of sensitivity and specificity. We searched the references of the identified studies to identify additional trials for inclusion.

### Data collection and analysis

#### Selection of studies

Two review authors (P.-C. C. and C.-H. C.) independently identified the studies for inclusion by screening the titles and abstracts. We sought full text for any references that were identified for potential inclusion by at least one of the authors. We made further selection for inclusion based on the full text. We have listed the full texts of references that we excluded with reasons for the exclusion in Appendix 2. We planned to list any ongoing trials identified primarily through World Health Organization International Clinical Trials Registry Platform for further follow-up. We resolved discrepancies through discussion.

### Data extraction and management

Two review authors (P.-C. C. and C.-H. C.) independently extracted the following data in Appendix 3.

1. Publication year.
2. Country in which the participants were recruited.
3. Inclusion and exclusion criteria.
4. Participant characteristics such as age, sex (male/female), and duration of symptoms of carpal tunnel syndrome.
5. Details of the intervention and treatment strategy that aimed to relieve symptoms and improve function (e.g., contents, dose, or approaches of injections).
6. Outcomes (clinical response, change in symptoms severity scale, and change in functional status scale).
7. Risk of bias.

We sought unclear or missing information by contacting the authors of the individual trials. We resolved any differences in opinion through discussion.

### Assessment of risk of bias in included studies

We followed the guidance given in the Cochrane Handbook for Systematic Reviews of Intervention to assess the risk of bias in included studies [29] . Specifically, we assessed the risk of bias in included studies for the following domains: random sequence generation (selection bias), allocation concealment (selection bias), blinding of participants and personnel (performance bias), blinding of outcome assessors (detection bias), incomplete outcome data (attrition bias), selective outcome reporting (reporting bias), and other bias (free of expertise bias) [30–36]. The risk of bias of each study was explicitly judged on each criterion and classified as 'low', 'high', or 'unclear'. The two review authors assessed the risk of bias of each study independently (P.-C. C. and C.-H. C.) and any disagreement was resolved through discussion to reach consensus.

### Measures of treatment effect

For dichotomous variables (clinical response), we calculated the odds ratio (OR) with 95 % credible interval (CrI). For continuous variables, such as change in symptom severity scale change and change in functional status scale change, we calculated the mean difference (MD) with 95 % CrI.

### Unit of analysis issues

The unit of analysis was the hands affected by carpal tunnel syndrome according to the intervention group to which they were randomly assigned.

### Dealing with missing data

We performed an intention-to-treat analysis [30] whenever possible. Otherwise, we used data that were available to us (e.g., a trial may have reported only per-protocol analysis results). For continuous outcomes, we imputed the standard deviation from *p* values according to guidance given in the *Cochrane Handbook for Systematic Reviews of Intervention* [29]. If the data were likely to be normally distributed, we used the median for meta-analysis when the mean was not available. If a study only reported the means and standard deviations for the baseline and follow-up measurements for each group, we needed to calculate the means and standard deviations for change in the outcome for these groups [31, 32].

### Assessment of heterogeneity

Clinical and methodological heterogeneity were assessed by carefully examining the characteristics and design of included studies. Major sources of clinical heterogeneity included age, sex, and duration of symptoms of carpal tunnel syndrome. Different study designs and risk of bias may contribute to methodological heterogeneity.

If substantial heterogeneity was identified - clinical, methodological, or statistical - we planned to explore and address heterogeneity in a subgroup analysis or meta-regression.

### Assessment of reporting biases

We planned to inspect a funnel plot asymmetry to explore reporting bias [32, 33]. It was the most common tool used to assess the presence of small study effects in a meta-analysis [34]. However, because of the estimate effects for different comparisons, there was no single reference line against which symmetry could be judged. To account for the fact, ‘comparison-adjusted’ funnel plot was suggested [34]. If the funnel plot suggested the presence of small study effects, we explored this further by sensitivity analysis.

### Data synthesis

We planned to apply classifications described in “Types of interventions” to categorize different injection approaches. We did not categorize different kinds or dose of corticosteroid into different interventions. Each category was broadly defined to encompass a relatively homogeneous group of interventions, although we anticipated that variations were noted in the way each local corticosteroid injections were applied. For example, different clinicians performed different technical skills of injections. These practice variations might be a source of heterogeneity. However, evidence was insufficient to suggest that these variations may affect the outcome. In local corticosteroid injections for carpal tunnel syndrome, a clinician typically chose one injection approach as described in “Types of interventions”, which was considered as a treatment strategy, or in terms of network meta-analysis, each unique treatment strategy could be defined as a ’node’. We planned to construct a network graph based on treatment strategies used in the studies that we identified.

We planned to perform conventional pairwise meta-analyses for all outcomes and comparisons, using a random-effects model [35] by STATA (StataCorp. 2013. *Stata Statistical Software: Release 13*. College Station, TX: StataCorp LP.), in accordance with recommendations of The Cochrane Collaboration [29]. We then performed a network meta-analysis for the outcomes (clinical response, change in symptom severity scale, and change in functional status scale). Network meta-analysis is a method of synthesizing information from a network of trials addressing the same question but involving different interventions [36, 37]. Network meta-analysis combines direct and indirect evidence across a network of randomized trials into a single effect size, and under certain assumptions it can increase the precision in the estimates while randomization is respected [38, 39]. We performed network meta-analyses within a Bayesian framework, assuming an equal heterogeneity parameter τ across all comparisons, and we accounted for correlations induced by multi-arm studies [40]. The analysis was performed using WinBUGS (MRC Biostatistics Unit, Cambridge, UK) (http://www.mrc-bsu.cam.ac.uk/software/bugs/the-bugs-project-winbugs/), and parameters were estimated based on 100000 iterations of the Markov chains after thinning them by retaining every 100th iteration; the codes and description of the methodology can be found at http://www.bristol.ac.uk/social-community-medicine/projects/mpes/mtc/. We used a normal prior with zero mean and variance one restricted to positive values for the common heterogeneity standard deviation τ and non-informative vague priors for all mean parameters, otherwise referred as treatment effects. As a measure that reflects ranking and the uncertainty, we used the Surface Under the Cumulative Ranking curve (SUCRA) as described by Salanti et al. [41]. This measure, expressed as percentage, showed the relative probability of an intervention being among the best options.

### Subgroup analysis and investigation of heterogeneity

We planned to perform the following subgroup analyses when at least one study was included in each subgroup.

1. Corticosteroid with different dose, potency, or duration.
2. Patients with different duration of symptoms.

We planned to use the Chi^2^ test to identify subgroup differences. We planned to consider a *p* value < 0.05 as statistically significant. However, we did not perform any of the above because of the few studies included in this network meta-analysis.

## Results

### Study selection

**Fig. 1:**
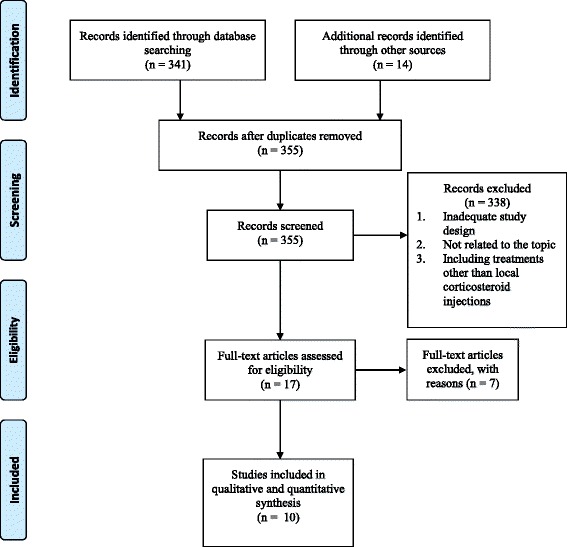
Flow diagram for literature search and identifications of articles for review

Figure 1 summarized the details of the study selection process and the reasons for exclusion. We identified 355 references through electronic searches and other sources. We excluded 338 inappropriate references through screening titles and reading abstracts. We retrieved 17 references for further assessment. Seven randomized controlled trials were excluded, and the reasons were described in Appendix 2 [42–48]. In total, 10 completed randomized clinical trials met the inclusion criteria [23–25, 27, 28, 49–53]. We chose the outcomes (clinical response, change in symptom severity scale, and change in functional status scale) assessed at short-term (before or near 8 weeks) follow-up period from these studies.

### Study description

The 10 trials included in the current systematic review were full reports published between 1999 and 2014. Table 1 summarized these studies. Because the symptom severity scale and the functional status scale in one study by Lee et al. [24]. was not the average of each item as defined by other studies, we divided the mean difference and the standard deviation by 11 (11 items in symptom severity scale of Boston Carpal Tunnel Questionnaire) for the change in symptom severity scale and divided the mean difference and the standard deviation by 8 (8 items in functional status scale of Boston Carpal Tunnel Questionnaire) for the change in functional status scale.

There were different dose of methylprednisolone used in one trial [50], and we adopted the 40 mg group compared to the placebo group in this meta-analysis. In addition, there were 3 different follow-up periods in the trial by Habib et al. [23], we chose the outcomes assessed at 6 weeks.

**Table 1:** Summary of included studies and patient characteristics

Study	Treatment strategies (contents/approach)	N	Age (years)	Sex (M/F)	Duration of symptoms (months)	Follow-up period (weeks)	Clinical response	MD of SSS (SD)	MD of FSS (SD)
Lee et al. 2014	G1: 40 mg TCA/PI	25	50.3	2/13	7.6	4	N/A	−0.63 (0.70)	−0.02 (0.75)
	G2: 40 mg TCA/UInar-O	24	52.6	0/14	9.4	4	N/A	−0.73 (0.55)	−0.35 (0.78)
	G3: 40 mg TCA/UInar-I	26	55.2	1/14	8.9	4	N/A	−1.16 (0.62)	−0.38 (0.70)
Makhlouf et al. 2014	G1: 80 mg TCA/PI	40	52.2	8/32	unknown	2	19/40	NA	NA
	G2: 80 mg TCA/Ulnar-I	37	45.7	2/35	unknown	2	34/37	NA	NA
Ustun et al. 2013	G1: 40 mg MTP/PI	23	42.7	1/22	10.2	6	N/A	−0.95 (0.63)	−1.16 (0.94)
	G2: 40 mg MTP/Ulnar-O	23	46.0	4/19	16.8	6	N/A	−1.27 (0.61)	−1.15 (0.68)
Atroshi et al. 2013	G1: placebo	37	49	9/28	14% ≦ 12 months	5	N/A	−0.47 (0.60)	N/A
	G2: 40 mg MTP/PI	37	44	10/27	27% ≦ 12 months	5	N/A	−1.33 (0.98)	N/A
Karadas et al. 2012	G1: placebo	30	48.4	2/17	9.9	8	N/A	−0.03 (0.52)	−0.03 (0.54)
	G2: 40 mg TCA/PI	30	46.4	3/17	9.5	8	N/A	−0.30 (0.69)	−0.15 (0.92)
Peters et al. 2010	G1: placebo	31	57.6	7/26	13	1	5/31	−0.29 (0.55)	0.14 (0.56)
	G2: 10 mg TCA/PI	35	56.5	9/27	26	1	17/35	−0.92 (0.71)	−0.58 (0.84)
Habib et al. 2006	G1: 35 mg of MTP/PI	21	43.3	4/17	5.5	6	15/21	N/A	N/A
	G2: 12 mg of MTP/DI	21	41	5/16	6	6	14/21	N/A	N/A
Armstrong et al. 2004	G1: 6 mg BMT/PI	43	51.9	8/35	61% > 1 year	2	30/43	−0.78 (0.80)	−0.64 (0.87)
	G2: placebo	38	51.2	10/28	66% > 1 year	2	13/38	−0.19 (0.62)	−0.13 (0.44)
O’Gradaigh et al. 2000	G1: 100 mg HC/PI	32	unknown	unknown	unknown	6	20/32	N/A	N/A
	G2: placebo	20	unknown	unknown	unknown	6	1/20	N/A	N/A
Dammers et al. 1999	G1: 40 mg MTP/PI	30	53	6/24	32	4	23/30	N/A	N/A
	G2: placebo	30	51	4/26	25	4	6/30	N/A	N/A

*Abbreviations*:

*N* number of treated hands; *TCA* triamcinolone acetonide; *MTP* methylprednisolone; *HC* hydrocortisone; *BMT* betamethasone; *SSS* symptom severity scale; *FSS* functional status scale; *DI* distal approach corticosteroid injection; *PI* proximal approach corticosteroid injection; *Ulnar-I* ultrasound-guided in-plane injection; *Ulnar-O* ultrasound-guided out-plane injection; *MD* mean difference; *SD* standard deviation

### Quality of studies

**Fig. 2:**
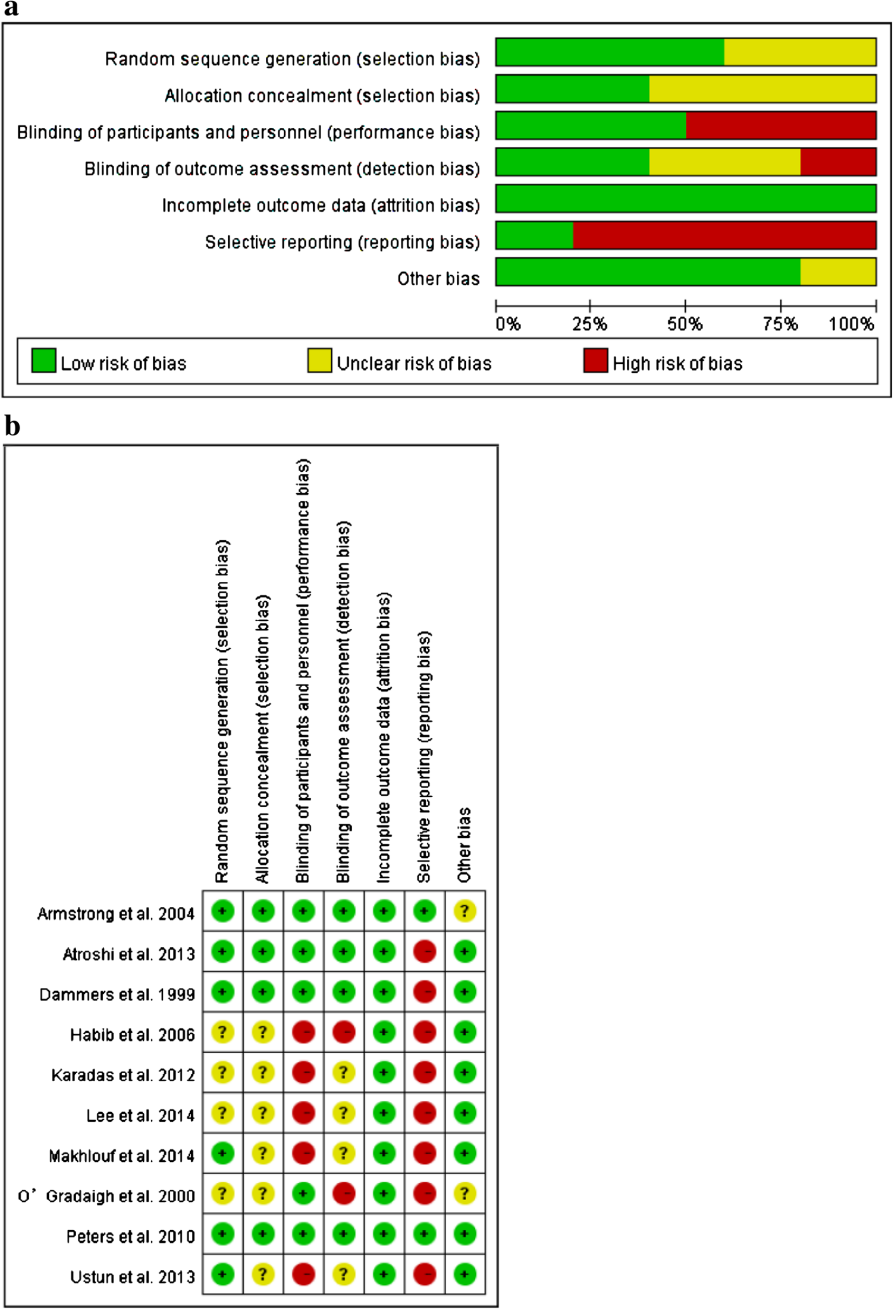
Risk of bias (**a**) graph and (**b**) summary: review authors’ judgements about each risk of bias item

Figure 2 showed the quality of the included studies. 10 studies described as randomized, 6 of which clearly described their randomize methods [25, 27, 28, 49–51]. The allocation concealment methods were described in 4 studies [28, 49–51], and the remaining did not provide details about it. 4 studies employed double blinding (patient and outcome assessor blinding) [28, 49–51]. Only 2 studies provided all of the 3 outcomes (clinical response, change in symptom severity scale, and change in functional status scale) [28, 49]. For the part of other bias, we discussed the expertise bias, and only 2 studies did not provide the details [28, 53].

### Effects of interventions

#### Pairwise meta-analysis (direct comparisons)

**Fig. 3:**
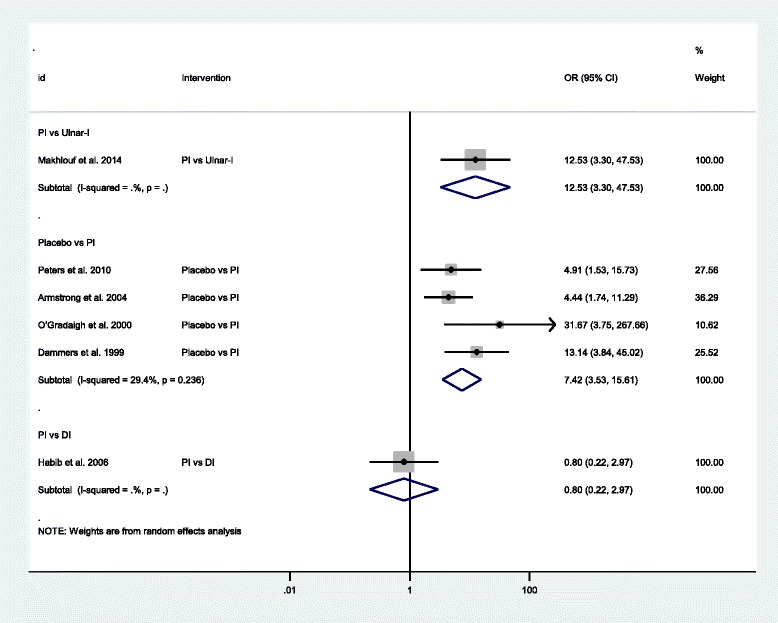
Forest plot of the standard pair-wise meta-analysis for clinical response of local corticosteroid injections for carpal tunnel syndrome. Abbreviations: *OR* odds ratio, *CI* confidence interval, *DI* distal approach corticosteroid injection, *PI* proximal approach corticosteroid injection, *Ulnar−I* ultrasound-guided in-plane approach

**Fig. 4:**
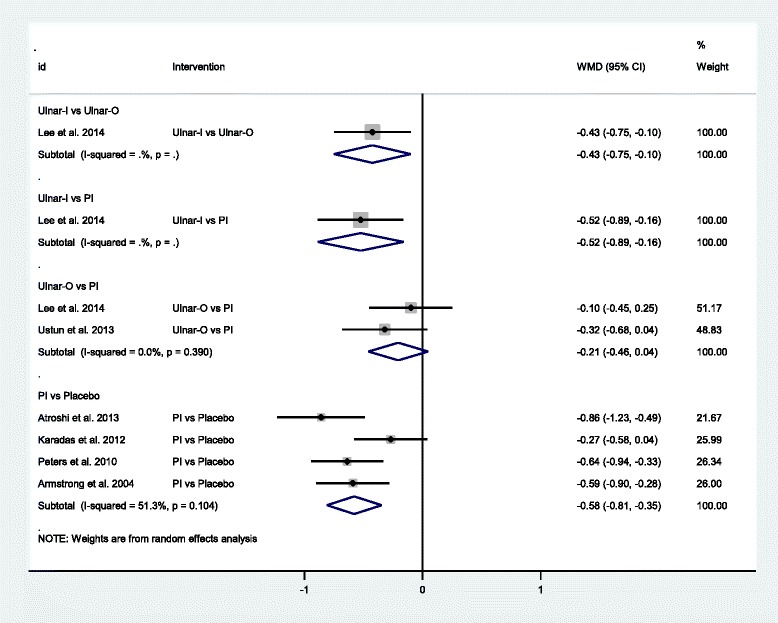
Forest plot of the standard pair-wise meta-analysis for change in symptom severity scale of local corticosteroid injections for carpal tunnel syndrome. Abbreviations: *WMD* weighted mean difference, *CI* confidence interval, *PI* proximal approach corticosteroid injection, *Ulnar−I* ultrasound-guided in-plane injection, *Ulnar−O* ultrasound-guided out-plane injection

**Fig. 5:**
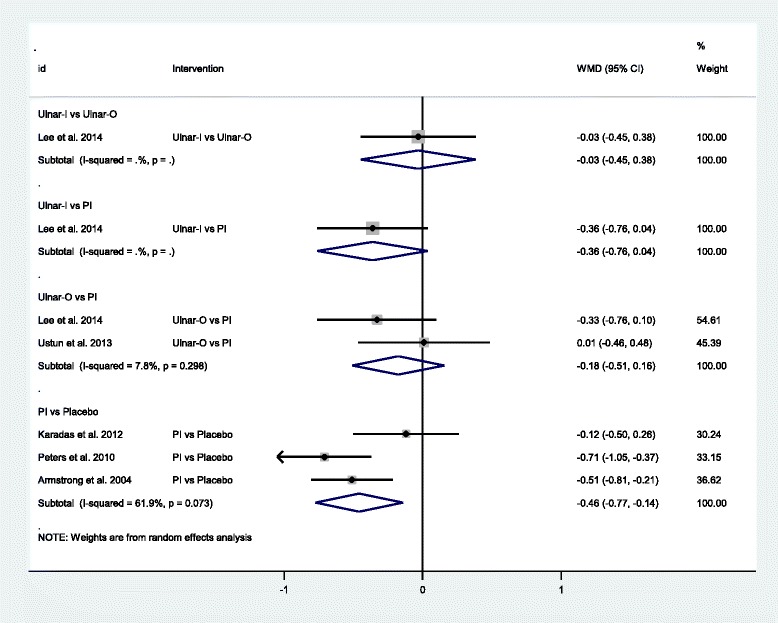
Forest plot of the standard pair-wise meta-analysis for change in functional status scale of local corticosteroid injections for carpal tunnel syndrome. Abbreviations: *WMD* weighted mean difference, *CI* confidence interval, *PI* proximal approach corticosteroid injection, *Ulnar−I* ultrasound-guided in-plane injection, *Ulnar−O* ultrasound-guided out-plane injection

Figures 3, 4, and 5 provided the pooled estimates for each major outcome.

1. Clinical response
  Six studies with 378 participants compared each treatment strategies [23, 26–28, 51, 53]. Local corticosteroid injections using the PI approach was effective for carpal tunnel syndrome as compared to placebo (OR = 7.42, 95 % CI 3.53 to 15.61, I^2^ = 29.4 %) [28, 49, 51, 53]. In one study [27], local corticosteroid injections using the Ulnar-I approach showed better clinical response than by the PI approach (OR = 12.53, 95 % CI 3.30 to 47.53). However, no statistical difference was found between the PI approach and the DI approach (OR = 0.80, 95 % CI 0.22 to 2.97) [23].
2. Change in symptom severity scale
  Six studies with 402 participants compared each treatment strategies [24, 25, 28, 49, 50, 52]. It appeared that local corticosteroid injections using the PI approach was effective for carpal tunnel syndrome as compared to placebo (MD = −0.58, 95 % CI −0.81 to −0.35, I^2^ = 51.3 %) [28, 49, 50, 52]. In one study [24], local corticosteroid injections using the Ulnar-I approach was better than using the Ulnar-O or the PI approach respectively (MD = −0.43, 95 % CI −0.75 to −0.10; MD = −0.52, 95 % CI −0.89 to −0.16). Local corticosteroid injections using the Ulnar-O approach and using the PI approach demonstrated similar effect (MD = −0.21, 95 % CI −0.46 to 0.04, I^2^ = 0.0 %) [24, 25].
3. Change in functional status scale
  Five studies with 328 participants compared each treatment strategies [24, 25, 28, 49, 52]. Similarly, local corticosteroid injections using the PI approach was also effective for carpal tunnel syndrome as compared to placebo (MD = −0.46, 95 % CI −0.77 to −0.14, I^2^ = 61.9 %) [28, 49, 52]. However, there was no statistical significance between the Ulnar-I and the Ulnar-O, the Ulnar-I and the PI, and the Ulnar-O and the PI (MD = −0.03, 95 % CI −0.45 to 0.38; MD = −0.36, 95 % CI −0.76 to 0.04; MD = −0.18, 95 % CI −0.51 to 0.16, I^2^ = 7.8 %) [24, 25].

### Network meta-analysis (combination of direct and indirect comparisons)

**Fig. 6:**
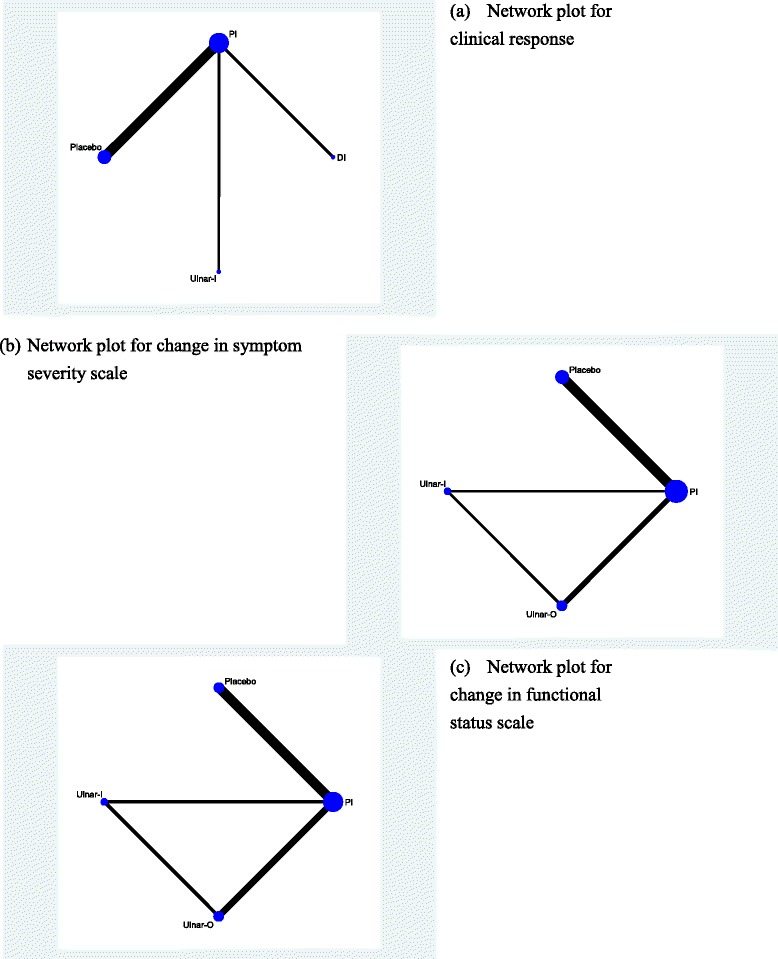
Network plots of the treatments for (**a**) clinical response, (**b**) change in symptom severity scale (**c**) change in functional status scale

Figure 6 showed the network plots of the treatments for clinical response, change in symptom severity scale, and change in functional status scale at short-term follow-up period. Any two nodes connected by the line represented direct comparisons in the trials. The thickness of the line was proportional to the number of comparisons included in the network, and the width of the circle was proportional to the number of studies involving the specific treatment.

**Fig. 7:**
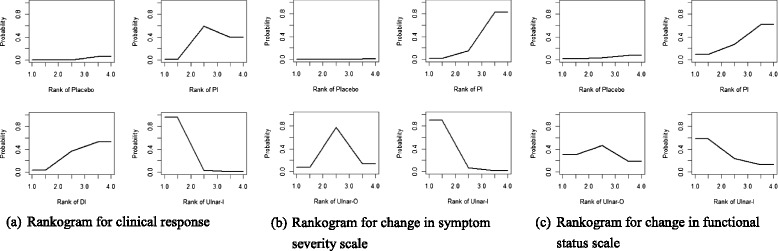
Ranking of treatment strategies based on probability of their effects on (**a**) clinical response (**b**) change in symptom severity scale (**c**) change in functional status scale

Table 2 reported the results of network meta-analysis, including summary posterior ORs or MDs with their 95 % credible intervals of all treatment strategies and SUCRA values expressed as a percentage. Figure 7 was the rankograms to show the rank probabilities.

**Table 2:** Summary results of network meta-analysis: posterior ORs or MDs with their 95% credible intervals of all treatment strategies and SUCRA values

	Clinical response versus placebo	
	Median OR (95% CrI)	SUCRA
Ulnar-I	128.30 (9.76 to 2299.00)	98%
DI	7.00 (0.53 to 118.80)	46%
PI	8.85 (3.00 to 33.15)	54%
	Change in symptom severity scale versus placebo	
	Median MD (95% CrI)	SUCRA
Ulnar-I	−1.16 (−1.95 to −0.38)	95%
Ulnar-O	−0.78 (−1.43 to −0.16)	64%
PI	−0.58 (−0.95 to −0.22)	40%
	Change in functional status scale versus placebo	
	Median MD (95% CrI)	SUCRA
Ulnar-I	−0.74 (−2.00 to 0.52)	78%
Ulnar-O	−0.63 (−1.67 to 0.43)	67%
PI	−0.46 (−1.11 to 0.21)	48%

*Abbreviations*:

*OR* odds ratio; *MD* mean difference; *CrI* credible interval; *SUCRA* surface under the cumulative ranking curve; *DI* distal approach corticosteroid injection; *PI* proximal approach corticosteroid injection; *Ulnar-I* ultrasound-guided in-plane injection; *Ulnar-O* ultrasound-guided out-plane injection

1. Clinical response
  Six studies with 378 participants were available [23, 27, 28, 49, 51, 53]. Local corticosteroid injections using the Ulnar-I approach might be the best choice among these interventions (median OR versus placebo 128.30, 95 % CrI 9.76 to 2299.00; SUCRA = 98 %), followed by local corticosteroid injections using the PI approach (median OR versus placebo 8.85, 95 % CrI 3.00 to 33.15; SUCRA = 54 %) and local corticosteroid injections using the DI approach (median OR versus placebo 7.00, 95 % CrI 0.53 to 118.80; SUCRA = 46 %).
2. Change in symptom severity scale
  These results were provided in six studies with 402 participants [24, 25, 28, 49, 50, 52]. It appeared that local corticosteroid injections using the Ulnar-I approach was the most effective among these interventions (median MD versus placebo −1.16, 95 % CrI −1.95 to −0.38; SUCRA = 95 %), followed by local corticosteroid injections using the Ulnar-O approach (median MD versus placebo −0.78, 95 % CrI −1.43 to −0.16; SUCRA = 64 %) and local corticosteroid injections using the PI approach (median MD versus placebo −0.58, 95 % CrI −0.95 to −0.22; SUCRA = 40 %).
3. Change in functional status scale
  Five studies with 328 participants provided the data [24, 25, 28, 49, 52]. The most effective intervention might be local corticosteroid injections using the Ulnar-I approach (median MD versus placebo −0.74, 95 % CrI −2.00 to 0.52; SUCRA = 78 %), followed by local corticosteroid injections using the Ulnar-O approach (median MD versus placebo −0.63, 95 % CrI −1.67 to 0.43; SUCRA = 67 %) and local corticosteroid injections using the PI approach (2; SUCRA = 48 %).

### Subgroup analysis and meta-regression

Subgroup analysis was not performed because of the paucity of data.

After reviewing Table 1, we came to the understanding that most cases were categorized as chronic carpal tunnel syndrome. However, we could not calculate the effect of this covariate owing to paucity of data. Likewise, various doses, potencies, and durations of corticosteroids were used for local corticosteroid injections in many studies and these also served as important covariates, but we could not calculate the effects of these covariates.

### Reporting bias

**Fig. 8:**
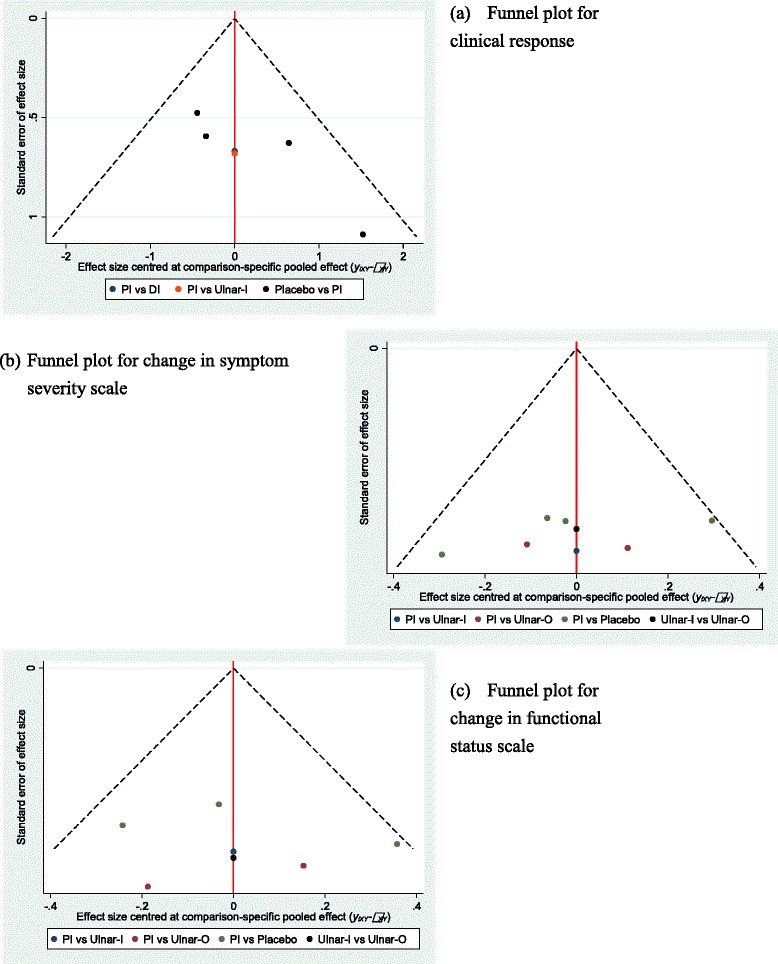
Comparison-adjusted funnel plots for (**a**) clinical response (**b**) change in symptom severity scale (**c**) change in functional status scale

The comparison-adjusted funnel plots for clinical response, change in symptom severity scale, and change in functional status scale, were shown in Fig. 8. Because sparse spots scattered on the funnel plots, it was hard to judge if any asymmetry existed. Therefore, it was likely to present reporting bias in this network meta-analysis.

## Discussion

This was the first network meta-analysis comparing local corticosteroid injections using different injection approaches for carpal tunnel syndrome. A total of 10 studies were included in the final pairwise meta-analysis and network meta-analysis. In the pairwise meta-analysis, we could know that local corticosteroid injections using the PI approach was better than placebo for clinical response, change in symptom severity scale, and change in functional status scale at short-term follow-up period. Local corticosteroid injections using the Ulnar-I approach was more effective than local corticosteroid injections using the PI approach for clinical response, and it was also more effective than local corticosteroid injections using the Ulnar-O or the PI approach for change in symptom severity scale. There was no statistical difference among the three groups: the PI approach versus the DI approach for clinical response, the Ulnar-O approach versus the PI approach for symptom severity scale, and the Ulnar-I approach versus the Ulnar-O approach versus the PI approach for change in functional status scale. In the network-meta-analysis, the results were similar to the results of the pairwise meta-analysis, but some differences were noted. It appeared that local corticosteroid injections using the Ulnar-I approach was the most effective for clinical response, change in symptom severity scale, and change in functional status scale at short-term follow-up period.

The primary goal of local corticosteroid injections using different injection approaches was to increase the clinical response and to improve symptomatic severity or functional status of hands affected by carpal tunnel syndrome. There was no significant difference in the severity of carpal tunnel syndrome among participants receiving local corticosteroid injections because we included participants with mild to moderate degree of carpal tunnel syndrome.

Local corticosteroid injections are generally practiced as conservative treatments for carpal tunnel syndrome. Ultrasound-guided injections are also being gradually well received by many clinicians [24, 25]. To sum up the results from the pairwise and network meta-analysis, we could deduce that:

1. Local corticosteroid injections were more effective than placebo for clinical response, change in symptom severity scale, and change in functional status scale at short-term follow-up period.
2. For injection approaches, the Ulnar-I approach might be more effective than the Ulnar-O approach or blind injection (including the PI and the DI approach).

Carpal tunnel syndrome was caused by increased carpal tunnel pressure, while the precise etiology was unknown. Experimental evidence suggested that anatomic compression or inflammation were possible mechanisms. [54] Through the first deduction from this meta-analysis, we favored inflammation as the major etiology of carpal tunnel syndrome for most patients. The Ulnar-I approach was recently proposed [55], and the first randomized controlled trial was completed by Lee et al. [24]. Although this injection approach seemed to be the most effective, small study effect should not be ignored. Future studies were still necessary to prove its effectiveness.

The statistical model of network meta-analysis not only provided the results of direct comparisons but also incorporated indirect comparisons that were rarely compared in the previous head-to-head studies. The strength of our study was that a practical and complete picture for the tendency of various injection approaches for the major outcomes of carpal tunnel syndrome. In addition, we also presented the probabilities of ranking for all these treatment strategies by using the Bayesian approach. The results of the probabilities of ranking could help the clinicians to choose better decisions for treatment.

Our study nonetheless had some limitations. Firstly, we did not analyze the long-term treatment effects because only few studies depicted these outcomes [44, 50, 51]. High dropout rates or referrals to surgical interventions after treatment failure were the main concerns [56, 57]. Future efforts on the long-term effects were still required. Secondly, we did not analyze the rates for adverse events due to various severity in each clinical trials. Comparing the rates for adverse events of each treatment strategies were an essential part of patient safety in recent years. Standardization of the report for adverse events of local injections might be a good solution, and the similar concepts have been mentioned in some articles [58–60]. Thirdly, although local corticosteroid injections using the Ulnar-I approach showed the highest probability of being the best choice for clinical response, change in symptom severity scale, and change in functional status scale at short-term follow-up period, small study effect should be kept in mind and the result should be interpreted cautiously in the clinical practice. Fourthly, one study [53] reported low clinical response for the treatment arms of placebo because no injection was performed, which could bias the results by affecting the actual outcomes of the participants in the trial [61]. Therefore, this could lead to increased uncertainty in the comparisons of multiple treatments within a network meta-analysis. This could account for wide range of incredible intervals for several treatment comparisons, in which the clinical response of the treatment arms for those trials was pretty low. Finally, the effects of covariates on our meta-analysis, such as characteristics of participants, duration of symptoms, various dose, potency, or duration of corticosteroids, or methodological quality, should be considered. Nevertheless, meta-regression of subgroup analysis could not be performed in our meta-analysis due to paucity of data. Further studies in the future were necessary to clarify these influences on the treatment outcomes.

## Conclusion

According to our analyses, the Ulnar-I approach was the most effective treatment for clinical response, change in symptom severity scale, and change in functional status scale among the injection approaches for carpal tunnel syndrome.

## Abbreviations

PI: Proximal corticosteroid injection
DI: Distal corticosteroid injection
Ulnar-O: Ultrasound-guided out-plane injection
Ulnar-I: Ultrasound-guided in-plane injection
CrI: Credible interval
SUCRA: Surface under the cumulative Ranking curve
