# Erratum to: A Bayesian network meta-analysis: Comparing the clinical effectiveness of local corticosteroid injections using different treatment strategies for carpal tunnel syndrome

**DOI:** 10.1186/s12891-015-0850-5

**Published:** 2015-12-22

**Authors:** Po-Cheng Chen, Ching-Hui Chuang, Yu-Kang Tu, Chyi-Huey Bai, Chieh-Feng Chen, Mei-Yun Liaw

**Affiliations:** Department of Rehabilitation, Kaohsiung Chang Gung Memorial Hospital, Kaohsiung, Taiwan; Department of Nursing, Kaohsiung Chang Gung Memorial Hospital, No.123, Dapi Road, Niaosong District, Kaohsiung, 83301 Taiwan; Institute of Epidemiology and Preventive Medicine, College of Public Health, National Taiwan University, Taipei, Taiwan; School of Public Health, Taipei Medical University, Taipei, Taiwan; Division of Plastic Surgery, Department of Surgery, Wan Fang Hospital, Taipei Medical University, Taipei, Taiwan; Cochrane Taiwan, Taipei Medical University, Taipei, Taiwan; School of Nursing, Chang Gung University of Science and Technology, Chiayi, Taiwan

## Erratum

During production of the original article [1], an oversight occurred which resulted in the omission of the Figures files from the published item. The original article has now been updated to include Figs. 1, 2, 3, 4, 5, 6, 7 and 8, as they appear below.

**Fig. 1:**
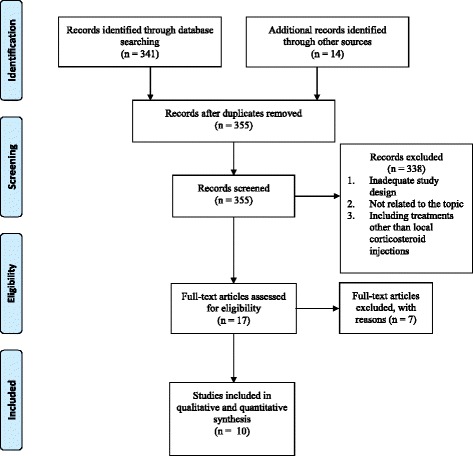
Flow diagram for literature search and identifications of articles for review

**Fig. 2:**
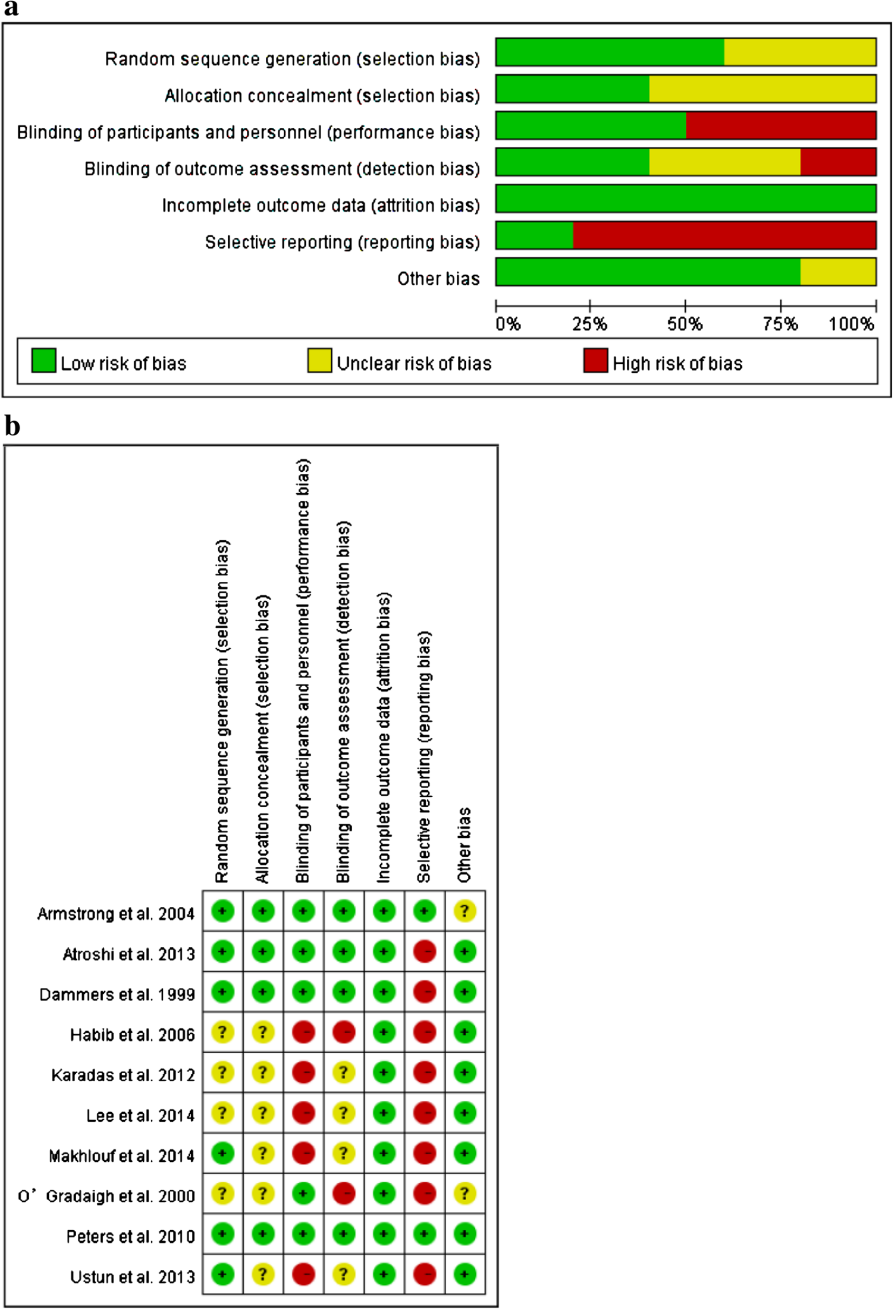
Risk of bias (**a**) graph and (**b**) summary: review authors’ judgements about each risk of bias item

**Fig. 3:**
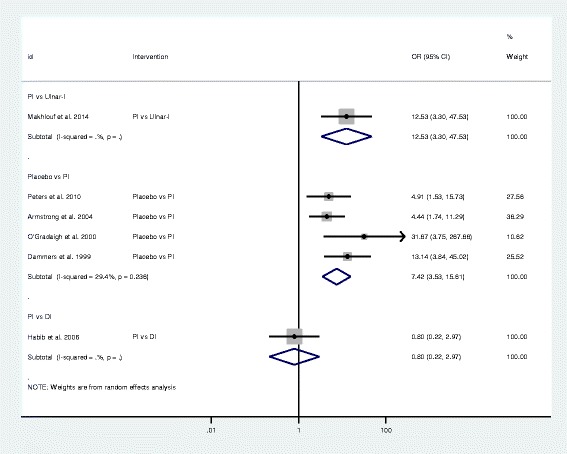
Forest plot of the standard pair-wise meta-analysis for clinical response of local corticosteroid injections for carpal tunnel syndrome. Abbreviations: OR = odds ratio, CI= confidence interval, DI = distal approach corticosteroid injection, PI = proximal approach corticosteroid injection, Ulnar-I = ultrasound-guided in-plane approach

**Fig. 4:**
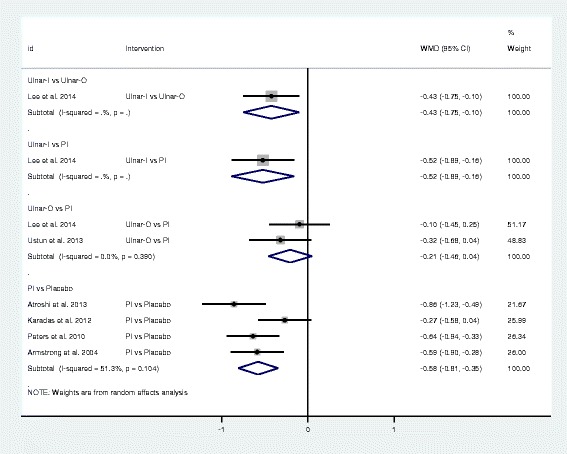
Forest plot of the standard pair-wise meta-analysis for change in symptom severity scale of local corticosteroid injections for carpal tunnel syndrome. Abbreviations: WMD = weighted mean difference, CI= confidence interval, PI = proximal approach corticosteroid injection, Ulnar-I = ultrasound-guided in-plane injection, Ulnar-O = ultrasound-guided out-plane injection

**Fig. 5:**
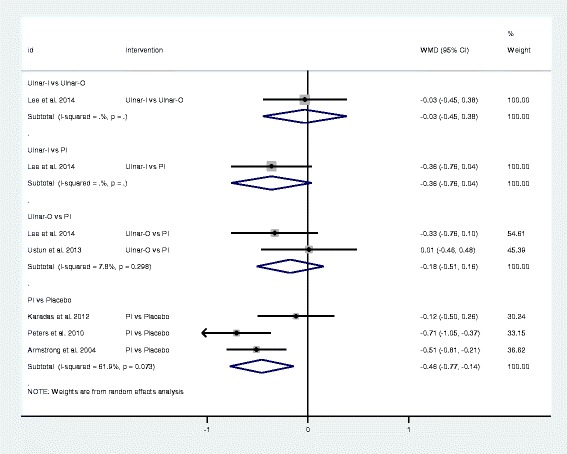
Forest plot of the standard pair-wise meta-analysis for change in functional status scale of local corticosteroid injections for carpal tunnel syndrome. Abbreviations: WMD = weighted mean difference, CI= confidence interval, PI = proximal approach corticosteroid injection, Ulnar-I = ultrasound-guided in-plane injection, Ulnar-O = ultrasound-guided out-plane injection

**Fig. 6:**
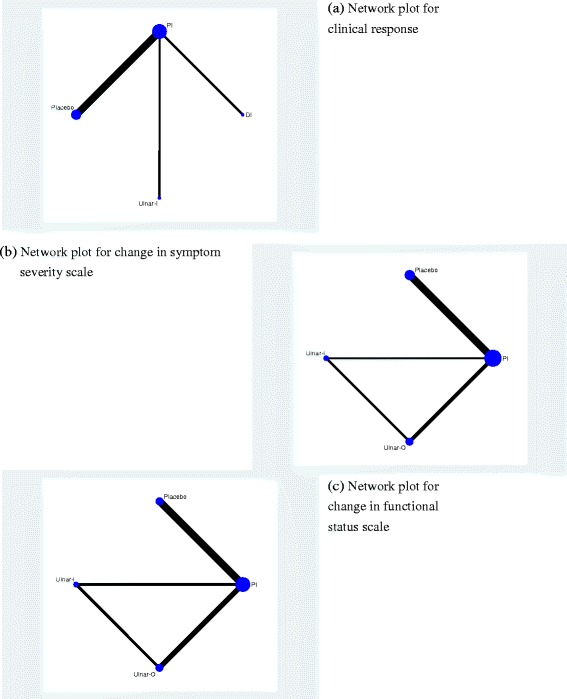
Network plots of the treatments for (**a**) clinical response, (**b**) change in symptom severity scale (**c**) change in functional status scale

**Fig. 7:**
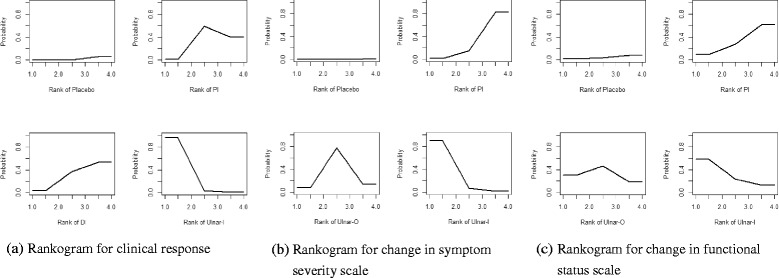
Ranking of treatment strategies based on probability of their effects on (**a**) clinical response (**b**) change in symptom severity scale (**c**) change in functional status scale

**Fig. 8:**
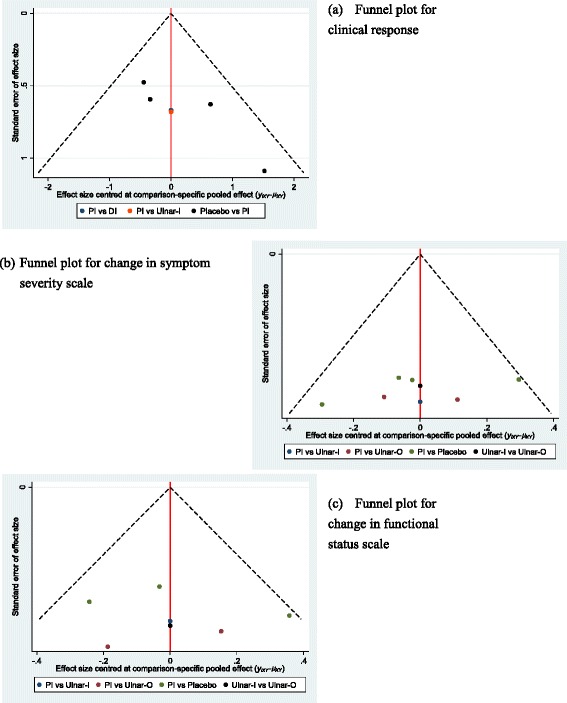
Comparison-adjusted funnel plots for (**a**) clinical response (**b**) change in symptom severity scale (**c**) change in functional status scale
